# Association between Timing and Duration of Adjuvant Chemotherapy and Colorectal Cancer Survival in Korea, 2011-2014: A Nationwide Study based on the Health Insurance Review and Assessment Service Database

**DOI:** 10.7150/jca.71141

**Published:** 2022-05-01

**Authors:** Jin Hwa Choi, Ji Sung Lee, Sun Kyung Baek, Jong Gwang Kim, Tae Won Kim, Seung Kook Sohn, Mi Yeon Kang, Sang-Cheol Lee, In Gyu Hwang

**Affiliations:** 1 Department of Radiation Oncology, Chung-Ang University Hospital, Seoul, South Korea.; 2 Clinical Research Center, Asan Medical Center, Seoul, South Korea.; 3 Kyung Hee University Medical Center, Seoul, South Korea.; 4 Department of Oncology/Hematology, Kyungpook National University Medical Center, Daegu, South Korea.; 5 Department of Oncology, Asan Medical Center, University, Seoul, South Korea.; 6 Health Insurance Review and Assessment Service, Wonju, South Korea.; 7 Quality Assessment Management Division, Health Insurance Review and Assessment Service, Wonju, South Korea.; 8 Soonchunhyang University Cheonan Hospital, Cheonan, South Korea.; 9 Department of Internal Medicine, Chung-Ang University College of Medicine, Seoul, South Korea.

**Keywords:** colorectal cancer, adjuvant chemotherapy, timing, duration

## Abstract

**Background:** Population-based analyses of the treatment outcomes of colorectal cancer (CRC) in Asian countries are limited. Therefore, we conducted a nationwide study to assess the relationship between the timing and duration of adjuvant chemotherapy (AC) and survival in patients with CRC in South Korea.

**Methods:** Data on AC from the Health Insurance Review and Assessment Service Database (HIRA) were analyzed, and the survival of patients who underwent curative-intent surgical resection for CRC between 2011 and 2014 was investigated.

**Results:** From the HIRA data, 45,992 patients with stage II-III CRC were identified. Chemotherapy regimens were administered as follows: 10,640 (23.3%) received 5-fluorouracil and leucovorin/capecitabine (FL/CAP), 13,083 (28.7%) received FL/CAP plus oxaliplatin (FOLFOX/CAPOX), 299 (0.7%) received uracil and tegafur/doxifluridine (UFT/D), and 21,570 (47.3%) underwent surgery alone. Patients who did not receive AC had worse survival than those who received AC in both the colon and rectum groups (HR, 1.96, 95% CI, 1.85-2.07 and HR, 2.18, 95% CI, 2.01-2.37, respectively). Regarding patients with stage II-III CRC, AC initiation ≥ 2 months after surgery was associated with a significant decrease in overall survival (OS) (FL/CAP: HR, 1.82; 95% CI, 1.53-2.17 and FOLFOX/CAPOX: HR, 2.92; 95% CI, 2.47-3.45); however, the effects of UFT/D regimens were not statistically significant. For patients with stage II-III colon cancer, AC <3 months had lower OS (FL/CAP: HR, 3.72, 95% CI, 2.80-4.94; FOLFOX/CAPOX: HR, 2.15, 95% CI, 1.87-2.47; and UFT/D: HR, 1.74, 95% CI, 0.56-5.41). In terms of patients with stage II-III rectal cancer, AC <3 months, regardless of chemotherapy regimens, had a significant lower survival (FL/CAP: HR, 1.91, 95% CI, 1.66-2.20; FOLFOX/CAPOX: HR, 2.20, 95% CI, 1.75-2.77; and UFT/D: HR, 3.71, 95% CI, 1.45-9.44).

**Conclusions**: Postoperative time to initiation and duration of AC were associated with survival. Based on our results, initiating AC within 2 months after surgery and administering AC for >3 months can potentially have an OS benefit in patients with stage II-III CRC.

## Introduction

With 19.3 million new cases and 10 million cancer deaths worldwide in 2020, colorectal cancer (CRC) is the third most frequent cancer and second leading cause of cancer death 1. The incidence rate of CRC in South Korea is increasing, the highest among the five major cancers, and it was reported to be 11.7% and 9.5% in men and women, respectively, among the estimated new cancer cases by sex in 2020. In South Korea, CRC deaths have also increased steadily; thus, CRC is ranked the third cause of cancer death 2.

Patients with localized CRC at initial diagnosis constitute 80% of all CRC cases 3. Surgery is the mainstay treatment for localized CRC, and adjuvant treatment may be used to eradicate micrometastatic spread. Adjuvant chemotherapy (AC) has consistently been shown to decrease recurrence and improve survival after curative resection of CRC and is recommended for patients with stage III-IV CRC, stage II rectal cancer, and stage II colon cancer with obstruction, perforation, T4 tumors, or other high-risk features 4, 5. Chemotherapy drugs, such as fluoropyrimidine and oxaliplatin, have been proven effective and are recommended; however, these treatments are associated with short-term and long-term toxicity. There are still some issues with AC regarding the appropriate timing of chemotherapy initiation and reduced duration of AC.

Most population-based analyses of CRC that have been conducted so far have evaluated incidences or treatment patterns or analyzed the effect of clinical characteristics such as age or stage on survival rates 6, 7. In our previous study, we also demonstrated clinical characteristics and survival in CRC according to age and cancer stage based on the same large dataset as that used in this study 8. However, few population-based studies on CRC treatment outcomes have been conducted in Asian countries. Hence, in this observational cohort study, we conducted a nationwide study to assess the relationship between the timing and duration of AC and survival in patients with CRC in South Korea.

## Material and Methods

### Study subjects

In this retrospective cohort study, we used the Health Insurance Review and Assessment Service (HIRA) database to examine the association between the timing and duration of AC and CRC survival. The HIRA database comprises all medical-expense claim data of South Koreans. The HIRA, which operates under the Korean National Health Insurance Program, contains the health data of all Koreans (97% health insurance and 3% medical care) 9. Since all clinics and hospitals submit claims data for in- and out-patient care, including diagnoses (as defined by the International Classification of Diseases, 10th revision [ICD-10]), demographic characteristics, diagnostic or surgical procedures, prescriptions, comorbidities, and the medical costs of claims, to HIRA to obtain reimbursement of medical costs from the government, HIRA contains the medical billing data of the entire South Korean population. Patients aged ≥18 years diagnosed with CRC (codes: C18, C19, and C20) between 2011 and 2014 in the HIRA records were eligible for enrolment. To assess the relationship between the timing and duration of AC and survival in patients with CRC in South Korea, stage II and III patients who underwent curative-intent surgical resection for CRC within the HIRA database were included in this study.

Data, including sex, age, stage, primary site, histology, and chemotherapy regimen, were collected. Age was divided into two groups, with 70 years being the cutoff age. Primary sites were defined based on ICD-10 codes and classified into colon and rectum. Adjuvant treatments were verified using preoperative and postoperative radiotherapy or chemotherapy claims. AC was considered implemented if there was a claim within 6 months after surgery, whereas RT was considered implemented if there was a claim within 3 months before and after surgery. AC regimens were classified according to prescription details, and the timing of postoperative AC initiation was defined as the period between the surgical prescription date and AC initiation date. AC duration was confirmed to be the entire period in which the same AC regimen was prescribed.

Survival was indirectly assessed using patient death dates recorded in the HIRA database. The outcome variable was overall survival (OS), defined as the time from the date of diagnosis until either the date of death or the end of the study (until June 30, 2016). This study was approved by the Institutional Review Board of Soonchunhyang University Cheonan Hospital (IRB no. SCHCA-2016-07-008). The requirement for patient informed consent was waived due to the retrospective nature of our study.

### Statistical analysis

OS probability according to AC timing or duration was calculated using the Kaplan-Meier product-limit method. OS hazard ratios (HRs) and 95% confidence intervals (CIs) were analyzed using Cox proportional hazard models for the timing and duration of AC. All statistical analyses were conducted using SAS software (version 9.4; SAS Institute, Cary, NC, USA). All P values were two-sided, and statistical significance was set at P <0.05.

## Results

### Baseline characteristics

A total of 71,513 patients were enrolled, of which the number of stage II and III patients was 45,992 from 2011 to 2014. The median follow-up duration was 3.2 years (range, 0.003-5.5 years). Male patients constituted 59.5%. The median age of all patients was 66 years (range, 18-102 years). Patients with stage II and III accounted for 29.0% and 35.6%, respectively. According to ICD-10 code, 72.1% and 27.9% of patients were diagnosed with colon and rectal cancers, respectively. The histological subtype was identified in a limited number of 23,682 patients, and 96%, 3.7%, and 0.3% of patients had adenocarcinoma, mucinous adenocarcinoma, and other or signet-ring cell type, respectively. In patients with stage II-III, AC was administered to 52.7% of patients. Chemotherapy regimens were administered as follows: 10,788 (23.3%) received 5-fluorouracil and leucovorin/capecitabine (FL/CAP), 13,266 (28.7%) received FL/CAP plus oxaliplatin (FOLFOX/CAPOX), and 303 (0.7%) received uracil and tegafur/doxifluridine (UFT/D) (Table 1).

### Survival according to adjuvant treatment

The effect of adjuvant treatment on survival was analyzed in patients with stage II-III. The patients were analyzed by dividing them into two groups: colon (C18 & C19) and rectum (C20), according to the ICD-10. In the colon group, the 3-year survival probabilities of patients who received AC and those who did not receive AC were 90.2% and 82.2%, respectively. In the rectum group, the 3-year survival probabilities were 88.6% and 77.1% in patients who received AC and those who did not receive AC and 84.9% and 78.6% in patients who received RT and those who did not receive RT, respectively (Table 2). Patients who did not receive AC had worse 3-year survival probabilities than those who received AC in both the colon and rectum groups (HR, 1.96, 95% CI, 1.85-2.07 and HR, 2.18, 95% CI, 2.01-2.37, respectively). Patients who did not receive RT also exhibited worse 3-year survival probabilities than those who received RT in the rectum group (HR, 1.52, 95% CI, 1.35-1.71).

### Survival according to the timing of postoperative adjuvant chemotherapy initiation

**Figure 1:**
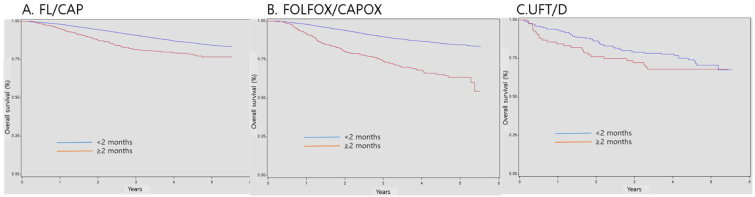
** Survival according to timing of postoperative adjuvant chemotherapy initiation.** FL/CAP: 5-fluorouracil and leucovorin/capecitabine; FOLFOX/CAPOX: FL/CAP plus oxaliplatin; UFT/D: uracil and tegafur/doxifluridine; CI: confidence interval; HR: hazard ratio.

Regarding patients with stage II-III CRC, the 3-year survival probabilities of those who started AC within 2 months of surgery were 90.9%, 89.8%, and 79.0% in the FL/CAP, FOLFOX/CAPOX, and UFT/D groups, respectively. However, the 3-year survival probabilities of patients who started AC months after surgery were 81.8%, 74.0%, and 72.3% in the FL/CAP, FOLFOX/CAPOX, and UFT/D groups, respectively. AC initiation at ≥ 2 months after surgery was associated with a significant decrease in OS (FL/CAP: HR, 1.82, 95% CI, 1.53-2.17 and FOLFOX/CAPOX: HR, 2.92, 95% CI, 2.47-3.45); however, the effects of UFT/D regimen were not statistically significant (Table 3 and Figure 1).

### Survival according adjuvant chemotherapy duration

**Figure 2:**
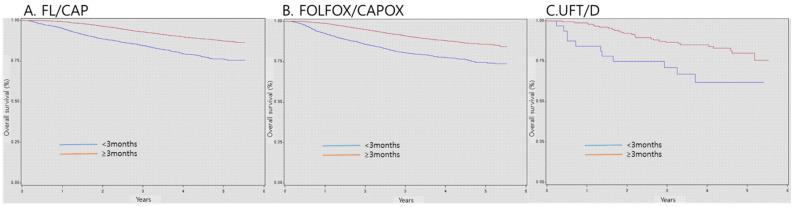
** Survival according to duration of adjuvant chemotherapy.** FL/CAP: 5-fluorouracil and leucovorin/capecitabine; FOLFOX/CAPOX: FL/CAP plus oxaliplatin; UFT/D: uracil and tegafur/doxifluridine; CI: confidence interval; HR: hazard ratio.

As regards patients with stage II-III CRC, the 3-year survival probabilities of patients who received AC for >3 months were 93.0%, 91.0%, and 86.7% in the FL/CAP, FOLFOX/CAPOX, and UFT/D groups, respectively. Nonetheless, the 3-year survival probabilities of patients who received AC for < 3 months were 84.6%, 80.5%, and 71.1% in the FL/CAP, FOLFOX/CAPOX, and UFT/D groups, respectively. Patients who received AC for < 3 months, regardless of chemotherapy regimens, exhibited a significantly lower survival (FL/CAP: HR, 2.28, 95% CI, 2.02-2.57; FOLFOX/CAPOX: HR, 2.16, 95% CI, 1.92-2.43; and UFT/D: HR, 2.36, 95% CI, 1.17-4.77) (Table 4 and Figure 2).

## Discussion

Population-based cancer survival, along with incidence and mortality, is one of the most important measures of the overall effectiveness of cancer care and control in a population. In addition, the trend of cancer survival facilitates the identification of changes in treatment outcomes according to changes in diagnosis and treatment 10. Previous population-based analyses of CRC treatment outcomes were based on the Western population 11, 12. Our study is the first nationwide study on CRC treatment outcomes in South Korea. Furthermore, our study provides comprehensive data regarding the relationship between postoperative AC timing and treatment duration and survival in patients with CRC.

In CRC patients, surgical resection is the only treatment that has curative potential. Postoperative AC has been proven to improve outcomes in high-risk patients since the 1990s and has become a standard treatment 13-15. Although benefit in stage II disease remains controversial, AC's benefits have predominantly been clearly demonstrated in stage III (node-positive) disease. Fluoropyrimidine-based chemotherapy is the standard adjuvant treatment. It decreases the risk of death by 10-15% in cases where fluorouracil monotherapy follows surgical resection and by 20-22% when it is used in combination with oxaliplatin 16. In our study, we also found that adjuvant treatment improves the survival rate of patients with stage II-III CRC. Postoperative omission of AC in patients with stage II-III CRC reduced OS with statistical significance, and omitting RT was associated with a significant decrease in the OS of rectal cancer patients.

Evidence regarding the optimal timing of AC after surgical resection or the ideal timing for adjuvant therapy after which treatment benefit increases is lacking. However, the importance of the interval between surgery and AC initiation has been clarified by several studies 17, 18. Colon cancer specific mortality was associated with time to AC initiation of >3 months (HR 1.48, 95% CI: 1.15-1.92) in 4000 patients with stage III colon cancer from the SEER database 18. A meta-analysis of 14 studies that included 17,645 patients revealed that an interval >8 weeks increased the relative risk of death (HR: 1.20, 95% CI: 1.15-1.26, p=0.001) 19. A retrospective study of 6620 patients with stage III colon cancer from the Netherlands Cancer Registry also revealed that initiating AC beyond 8 weeks was associated with decreased OS compared to initiation within 2 months (HR: 1.4 [1.21-1.68] at 9-10 weeks;: 1.3 [1.06-1.59] at 11-12 weeks; and 1.7 [1.23-2.23] at 13-16 weeks) 24. This population-based study was conducted to evaluate the relationship between AC timing and survival in patients receiving FL/CAP, FOLFOX/CAPOX, and UFT/D. AC 2 months after surgery was found to be significantly associated with worse OS in the FL/CAP and FOLFOX/CAPOX groups. Our results indicate that AC timing within 2 months after surgery has a significant OS benefit, regardless of AC regimen. Patients who start AC > 2 months after surgery potentially develop postoperative complications and comorbidities that may result in delayed AC, thus causing potential bias in this analysis. However, based on previous studies and our research, ideal AC initiation is recommended within 2 months after surgery. UFT plus leucovorin has been shown to be a convenient, well-tolerated, and effective treatment regimen for advanced CRC 20. Although UFT/D was less beneficial in this study, the results need to be interpreted with care because the number of samples using UFT/D was small compared to that of other regimens.

While studies on AC in both colon and rectal adenocarcinoma have been conducted, no anticancer agents have proven to be as effective as AC for colon cancer, except for fluoropyrimidine and oxaliplatin. In addition to the “National Surgical Adjuvant Breast and Bowel Project C-07” report 21, the “Multicenter International Study of Oxaliplatin/ 5FU-LV in the Adjuvant Treatment of Colon Cancer” trial 22 revealed that adding oxaliplatin to a regimen of FL significantly improved disease-free survival and OS, and 12 subsequent cycles of FOLFOX became the standard adjuvant regimen for stage III colon cancer treatment. Despite the efficacy of FOLFOX treatment for stage III colon cancer, oxaliplatin-induced toxicity has emerged as an important medical problem. In particular, oxaliplatin-induced neurotoxicity is cumulative dose-dependent and causes permanent peripheral neuropathy, which potentially exerts worse effects on patients' quality of life. Physicians have attempted to modify the AC schedule, including duration, to improve patient outcomes and decrease treatment-related toxicity. The “International Duration Evaluation of Adjuvant Chemotherapy” collaboration conducted a pooled analysis of six large randomized controlled trials that compared 3 versus 6 months of AC with FOLFOX or CAPOX. In the high-risk group (T4 and/or N2), 3 months of FOLFOX was inferior to 6 months of FOLFOX; nevertheless, 3 months of CAPOX was not inferior to 6 months of CAPOX. In the low-risk group (T1-3N1 patients), in addition to 3 months of FOLFOX, 3 months of CAPOX was also not inferior to 6 months of FOLFOX and 3 months of CAPOX 23. In this study, the relationship between AC duration and survival rate was evaluated by categorizing participants into 3 months before and after among those receiving FL/CAP, FOLFOX/CAPOX, and UFT/D, respectively. AC for <3 months was found to be significantly associated with worse OS in the FL/CAP, FOLFOX/CAPOX, and UFT/D groups. Our results indicate that >3 months' AC has a significant OS benefit, regardless of AC regimen.

Notwithstanding, this study has certain limitations. First, this was a retrospective study utilizing vast amounts of data from the existing HIRA database; thus, the potential for confounding, based on patient selection, could not be entirely eliminated. Moreover, high-risk factors for recurrence in stage II CRC, including perineural invasion, lymphovascular emboli, lymph node harvest less than 12, T4 stage, positive resection margin, obstruction, and perforation, were not fully available in the HIRA database. Therefore, this study also included stage II CRC patients without high-risk factors who are not candidates for AC. Second, some clinical variables were not available, and missing data related to histology and differentiation prevented a comparative analysis. Therefore, there could be other important confounding factors that should not be discarded. Third, this study included patients with both colon and rectal cancers. Although there are differences in the method of adjuvant treatment for these diseases, the inclusion of patients whose final stage merited AC according to the National Comprehensive Cancer Network guidelines minimized these differences in the present study. Nevertheless, this study was meaningful in that it compared results according to the treatment details of Korean patients with CRC using vast amounts of data of over 70,000 patients.

In conclusion, more than half of the patients with stage II-III CRC in South Korea underwent AC after surgery, and AC improved their survival. Postoperative time to initiation and duration of AC were associated with survival. In patients with stage II-III CRC, initiating AC within 2 months after surgery and administering AC for >3 months can potentially have an OS benefit.

## Figures and Tables

**Table 1:** Baseline characteristics

Characteristics	Total (n=45,592)
**Sex**	
Male	27,148 (59.5%)
Female	18,444 (40.5%)
**Age**	
Median (range)	66 (18-102)
18-70 years	27,738 (60.8%)
≥70 years	17,854 (39.2%)
**Stage**	
Stage II	20,471 (44.9%)
Stage III	25,121 (55.1%)
**Primary site (by ICD-10)**	
Colon (C18 & C19)	32,884 (72.1%)
Rectum (C20)	12,708 (27.9%)
**Histology (n=23,682)**	
Adenocarcinoma	22,723 (96.0%)
Mucinous adenocarcinoma	887 (3.7%)
Others, Signet-ring cell carcinoma, Unknown	72 (0.3%)
**Adjuvant chemotherapy**	
No	21,570 (47.3%)
FL/CAP	10,640 (23.3%)
FOLFOX/CAPOX	13,083 (28.7%)
UFT/D	299 (0.7%)

FL/CAP: 5-fluorouracil and leucovorin/capecitabine; FOLFOX/CAPOX: FL/CAP plus oxaliplatin; UFT/D: uracil and tegafur/doxifluridine.

**Table 2:** Survival according to adjuvant treatment

Adjuvant treatment	Total number of patients	Total number of events	Survival probability at 1 year, % (95% CI)	Survival probability at 3 years, % (95% CI)	Survival probability at 5 years, % (95% CI)	HR (95% CI)	P-value
**AC (colon)**							
Yes	16,220	1777	97.8 (97.6-98.1)	90.2 (89.8-90.7)	84.9 (84.2-85.7)	Ref	
No	16,664	3242	92.8 (92.4-93.2)	82.2 (81.5-82.8)	73.5 (72.5-74.4)	1.96 (1.85-2.07)	<.0001
**AC (rectum)**							
Yes	7802	1097	97.7 (97.4-98.1)	88.6 (87.9-89.3)	81.6 (80.5-82.7)	Ref	
No	4906	1290	91.0 (90.2-91.8)	77.1 (75.8-78.3)	65.2 (63.4-66.9)	2.18 (2.01-2.37)	<.0001
**RT (rectum)**							
Yes	6897	1091	96.7 (96.0-97.3)	84.9 (83.5-86.3)	76.7 (74.6-78.8)	Ref	
No	5811	1296	91.5 (90.4-92.5)	78.6 (77.0-80.2)	68.5 (66.2-70.7)	1.52 (1.35-1.71)	<.0001

AC: adjuvant chemotherapy; RT: radiotherapy; CI: confidence interval; HR: hazard ratio.

**Table 3:** Survival according to timing of postoperative adjuvant chemotherapy initiation

Adjuvant regimen	Timing of adjuvant chemotherapy	Total number of patients	Total number of events	Survival probability at 1 year, % (95% CI)	Survival probability at 3 years, % (95% CI)	Survival probability at 5 years, % (95% CI)	HR (95% CI)	P-value
FL/capecitabine	<2 months	9895	1076	98.2 (97.9-98.4)	90.9 (93.3-91.5)	84.5 (83.5-85.4)	Ref	<.0001
	≥ 2 months	745	139	95.2 (93.7-96.8)	81.8 (78.8-84.7)	76.8 (73.0-80.6)	1.82 (1.53-2.17)	
FOLFOX/CAPOX	<2 months	12,544	1427	97.9 (97.7-98.2)	89.8 (89.3-90.4)	84.6 (83.8-85.4)	Ref	<.0001
	≥ 2 months	539	152	91.6 (89.3-93.9)	74.0 (70.0-77.9)	63.6 (58.1-69.1)	2.92 (2.47-3.45)	
UFT/D	<2 months	216	54	93.6 (90.4-96.8)	79.0 (73.5-84.5)	70.7 (63.5-77.9)	Ref	0.2617
	≥ 2 months	83	26	84.5 (76.8-92.3)	72.3 (62.6-81.9)	68.0 (57.7-78.2)	1.31 (0.82-2.08)	

FL/CAP: 5-fluorouracil and leucovorin/capecitabine; FOLFOX/CAPOX: FL/CAP plus oxaliplatin; UFT/D: uracil and tegafur/doxifluridine; CI: confidence interval; HR: hazard ratio.

**Table 4:** Survival according to duration of adjuvant chemotherapy

Adjuvant regimen	Duration of adjuvant chemotherapy	Total number of patients	Total number of events	Survival probability at 1 year, % (95% CI)	Survival probability at 3 years, % (95% CI)	Survival probability at 5 years, % (95% CI)	HR (95% CI)	P-value
FL/capecitabine	<3 months	2461	472	95.2 (94.4-96.1)	84.6 (83.1-86.1)	76.4 (74.1-78.7)	2.28 (2.02-2.57)	<.0001
	≥ 3 months	8179	743	99.3 (99.1-99.5)	93.0 (92.4-93.6)	87.4 (86.4-88.4)	Ref	
FOLFOX/CAPOX	<3 months	1798	384	92.3 (91.1-93.6)	80.5 (78.6-82.4)	74.6 (72.1-77.2)	2.16 (1.92-2.43)	<.0001
	≥ 3 months	11,285	1195	98.8 (98.6-99.0)	91.0 (90.4-91.6)	85.7 (84.9-86.6)	Ref	
UFT/D	<3 months	51	23	84.4 (71.8-97.0)	71.1 (55.0-87.1)	61.9 (43.5-80.4)	2.36 (1.17-4.77)	0.0164
	≥ 3 months	248	57	98.7 (96.9-100.0)	86.7 (81.3-92.1)	80.0 (72.6-87.4)	Ref	

FL/CAP: 5-fluorouracil and leucovorin/capecitabine; FOLFOX/CAPOX: FL/CAP plus oxaliplatin; UFT/D: uracil and tegafur/doxifluridine; CI: confidence interval; HR: hazard ratio.

## Abbreviations

AC: adjuvant chemotherapy
CI: confidence interval
CRC: colorectal cancer
FL/CAP: 5-fluorouracil and leucovorin/capecitabine
FOLFOX/CAPOX: 5-fluorouracil and leucovorin/capecitabine plus oxaliplatin
HIRA: Health Insurance Review and Assessment Service
HR: hazard ratio
ICD-10: International Classification of Diseases, 10th revision
OS: overall survival
UFT/D: uracil and tegafur/doxifluridine
